# Advances in Improving Energy Efficiency of Fiber–Wireless Access Networks: A Comprehensive Overview

**DOI:** 10.3390/s23042239

**Published:** 2023-02-16

**Authors:** Josip Lorincz, Zvonimir Klarin, Dinko Begusic

**Affiliations:** 1 Faculty of Electrical Engineering, Mechanical Engineering and Naval Architecture (FESB), University of Split, R. Boskovica 32, 21000 Split, Croatia; 2 University of Applied Sciences Sibenik, Trg Andrije Hebranga 11, 22000 Sibenik, Croatia

**Keywords:** radio, access, networks, fiber, D-RAN, C-RAN, Mobile fronthaul, RoF, digitized RoF, analog RoF, NGFI, FiWi, NG–PON, energy efficiency, SDN, NFV, MEC

## Abstract

Due to the growing impact of the information and communications technology (ICT) sector on electricity usage and greenhouse gas emissions, telecommunication networks require new solutions which will enable the improvement of the energy efficiency of networks. Access networks, which are responsible for the last mile of connectivity and also for one of the largest shares in network energy consumption, are viable candidates for the implementation of new protocols, models and methods which will contribute to the reduction of the energy consumption of such networks. Among the different types of access networks, hybrid fiber–wireless (FiWi) networks are a type of network that combines the capacity and reliability of optical networks with the flexibility and availability of wireless networks, and as such, FiWi networks have begun to be extensively used in modern access networks. However, due to the advent of high-bandwidth applications and Internet of Things networks, the increased energy consumption of FiWi networks has become one of the most concerning challenges required to be addressed. This paper provides a comprehensive overview of the progress in approaches for improving the energy efficiency (EE) of different types of FiWi networks, which include the radio-and-fiber (R&F) networks, the radio-over-fiber networks (RoF), the FiWi networks based on multi-access edge computing (MEC) and the software-defined network (SDN)-based FiWi networks. It also discusses future directions for improving the EE in the FiWi networks.

## 1. Introduction

Climate change manifested by global warming caused by large amounts of greenhouse gas (GHG) emissions presents a serious issue that affects today’s society. This issue is contributed to by increased energy demand for powering today’s Information and Communication Technology (ICT) systems. This increased energy demand for ICT systems additionally poses an economic issue due to the increasing cost and consumption of energy. Therefore, improving the energy efficiency (EE) of ICT systems has become an important consideration, since addressing this issue can contribute to the global reduction of GHG emissions and operating expenditure (OPEX) costs of service providers and network owners.

Despite the ability to reduce GHG emissions in some real-life scenarios (e.g., transportation reduction and document dematerialization), ICT is becoming a major contributor to global energy consumption [1]. The EE in telecommunication networks, as one of the significant contributors to the energy consumption of the overall ICT industry, is attracting global attention due to its large contribution to increasing OPEX costs and carbon dioxide emissions. Depending on the usage scenario, it is projected that communication technology can contribute to an increase in global electricity usage between 8% and 51% by 2030 [2]. Today’s communication networks are dominated by wireless technologies (e.g., mobile cellular or wireless local/personal/access networks), and wireless traffic is expected to increase dramatically with the full deployment of the fifth-generation (5G) mobile network [3]. This is a consequence of the basic aim of the 5G technology, which is envisioned to support a massive number of connected devices through the Internet of Things (IoT) concept. This will result in usage scenarios where communication technology will be incorporated into every aspect of today’s society. To support an enormous number of devices, the 5G is designed as a heterogeneous network (HetNet) with the ultra-dense deployment of small base stations (BSs) operating in sub-6 GHz and millimeter-wave (mmWave) frequency bands and employing massive multiple-input multiple-output (mMIMO) transmission technologies. Hence, an increasing number of connected devices with a constant demand for higher data rates will support new services and applications, requiring an increase in the number of supporting network infrastructure elements, which negatively affects the EE of mobile networks (MNs). Radio access networks (RAN) and particularly BSs are the largest, in terms of number and power consumption, individual elements of the RAN, and consequently they add a significant contribution to the overall energy consumption of telecommunication networks. Therefore, the EE of RAN should be a key concern in the design and deployment of future MNs [4].

**Figure 1:**
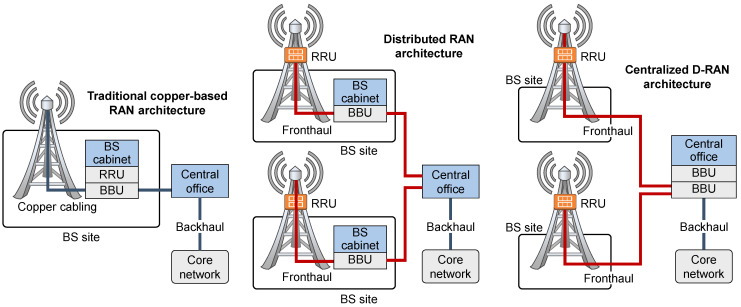
Comparison of traditional RAN and D-RAN architecture.

Due to the abovementioned necessity for satisfying implementation demands related to the simultaneous serving of higher numbers of users, offering larger data rates or ensuring a better quality of service, mobile network operators (MNOs) in past decades upgraded their radio access network (RAN) architectures. One of the main RAN architecture upgrades is related to the replacement of obsolete and overhauled traditional waveguide and coaxial-based BSs architecture (Figure 1) with those based on fiber–optic systems. Such architecture, through exploiting wired fiber–optic links for connecting physically spaced parts of BSs, establishes a completely new concept of data transmission in the RANs known as Distributed-RAN (D-RAN) (Figure 1). Introduced for third-generation (3G) mobile networks, the D-RAN architecture is characterized by modular BS design. This design decouples BS hardware into a remote radio unit (RRU) dedicated to the transmission and reception of wireless signals and a Baseband Unit (BBU) dedicated to baseband digital signal processing (Figure 1). Such D-RAN architecture, also known as Fiber to the Antenna (FTTA) architecture, creates two physically separated BS components that are connected with optical fiber at the location of the BS site, or up to a few hundred meters away.

The introduction of such modular BS design with fiber–optic cabling between BBU and RRU provides numerous advantages which primarily include higher bandwidth, reduced transmission losses and lower sensitivity to electromagnetic interference and noise. However, one additional positive aspect of such architecture is reducing the increased energy consumption of network equipment, since RRU can exploit the concept of natural air cooling, which reduces the energy needed for cooling overall BSs composed of BBU and RRU in the same cabinet (Figure 1).

Additionally, the introduction of optical communication between RRU and BBU in D-RAN becomes the basis for the development of the newest generation of RAN architectures, which are based on centralized wireless access networks known as Cloud-RAN (C-RAN). In the C-RAN, decupled BS RRU and BBU components are placed at different locations which can be up to tens of kilometers away (Figure 1). Locations are connected with fiber–optic technology which establishes a new RAN entity defined as a mobile fronthaul network (MFN). Although the realization of MFN based on the C-RAN architecture was initially introduced for fourth-generation (4G) mobile networks, the superior bandwidth capabilities and network scalability that C-RAN provides set the basis for the full exploitation of the C-RAN concept in the realization of the MFN of 5G networks and future sixth-generation (6G) mobile networks. Although most of the modern BSs currently use D-RAN architecture, the C-RAN can be viewed as the architectural evolution of the D-RAN and the C-RAN architecture and will be essential for the full practical deployment of the 5G and the future 6G RANs.

Besides implementation in mobile cellular networks, the integration of fiber optics and wireless technology in access networks through the combination of fiber and wireless technologies, also known as the fiber–wireless (FiWi) concept, presents an alternative cost-effective solution to the realization of access networks. FiWi access networks integrate the high capacity of optical fiber networks with the coverage and flexibility of wireless networks. They create a robust infrastructure for the development and deployment of current and future applications and services and they are considered to be the most promising option for the realization of next-generation access networks [5]. By using optical networks as the backhaul and wireless networks as the front-end, FiWi networks aim to provide straightforward access for users [6]. These networks are expected to meet the demands of future access networks, such as providing high bandwidth, reliability, low cost and flexibility [7]. Additionally, it has been reported that access networks account for approximately 70% of energy usage in the ICT industry, and as a result of an increasing number of communication devices and bandwidth rates, this percentage is likely to increase in the future [6]. Therefore, to decrease overall network energy consumption, it is crucial to develop energy-efficient architectures of access networks, and architecture based on the FiWi concept is seen as a promising contributor to this goal.

In a FiWi network, both radio-over-fiber (RoF) and radio-and-fiber (R&F) technologies are utilized. Radio-over-fiber (RoF) constitutes a popular communication system design that addresses the increasing bandwidth demand and enables optical and wireless integration in modern C-RAN systems. The radio-and-fiber (R&F) network, in contrast, is an approach based on connecting distributed radio transmitters (such as wireless local area network (WLAN) access points (APs)) and centralized WLAN controllers with fiber–optic networks. R&F networks have the advantage of building wireless local area network (WLAN)-based FiWi networks. These advantages are reflected in the possible coverage of larger areas without the limitations related to the optical back-end size, while the RoF network has a limited reach of deployed fiber imposed by the propagation delay in fiber. Using the same infrastructure for both wired and wireless services, FiWi networks can merge the traditionally separate optical and wireless access networks, thus leading to potential cost savings [8].

The high performance requirements of future 5G networks, including extremely low latency, uninterrupted user experience and high throughput and reliability, will necessitate the use of localized services that are within RANs located closer to mobile subscribers.

The multi-access edge computing (MEC) paradigm is exploited as a solution for offering services closer to mobile subscribers. Through telecom operators, information technology (IT) and cloud computing, the MEC aims to bring cloud services to the users directly from the network edge [9]. However, the concept of MEC over FiWi networks must address not only the 5G network realization challenges, but it must also address unique challenges related to the integration of the MEC concept with existing wired and wireless infrastructures, and managing resources effectively in the context of backhaul and RAN coordination [9]. Furthermore, a software-defined network (SDN) architecture which uses a centralized controller to manage networks is a revolutionary approach that has the potential to greatly improve the control and management of FiWi access networks, and thus increase the EE of the entire FiWi network [10]. Therefore, in this paper, EE aspects of the FiWi networks with particular emphases on the RoF, R&F, MEC and SDN technologies were overviewed.

The paper is further structured as follows: the overview of the FiWi access networks is presented in Section 2. In Section 3, the EE challenges of R&F networks are discussed. The energy-efficient RoF solutions are elaborated in Section 4, with an emphasis on the impact of the C-RAN network architecture on the EE. The EE solutions for MEC-based FiWi access networks are reviewed in Section 5. In Section 6, the SDN-based energy-saving schemes in FiWi networks are elaborated. Section 7 discusses the results of performed analyses dedicated to improving FiWi networks EE. Finally, conclusion remarks related to the review of EE methods for FiWi networks are given in Section 8.

## 2. Fiber–Wireless Broadband Access Network

Merging optical fiber and wireless technologies in broadband access networks by leveraging the advantages of both technologies presents a viable solution to support the increasing bandwidth demands of future applications [11,12]. The concept of FiWi access networks is based on the seamless integration of the mobility and coverage offered by wireless networks and the high bandwidth and better stability offered by optical technology. In comparison with traditional access networks, FiWi access networks can be considered as an alternative or transitional solution that provides high data rates more cost-effectively with the desired quality of service (QoS).

### 2.1. Architecture of FiWi Networks

**Figure 2:**
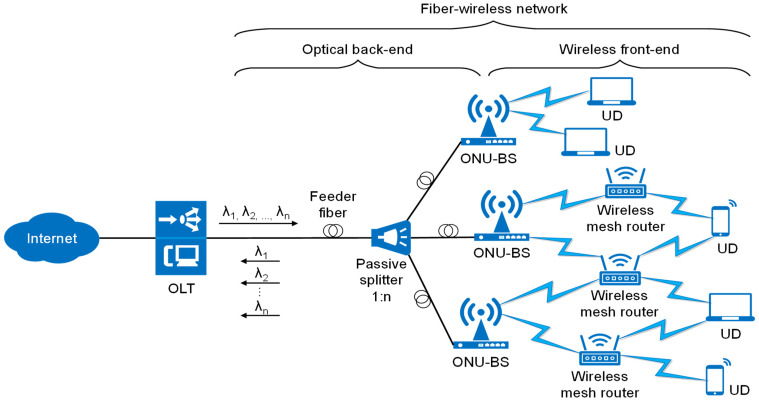
Illustration of R&F integration between optical and wireless networks.

The most common architecture of FiWi access networks comprises optical and wireless network domains. Ethernet passive optical networks (EPONs) or gigabit passive optical networks (GPONs) are commonly used in the optical segment of the network. In the wireless domain, a wireless point-to-point or mesh network (WMN) is operated according to the IEEE 802.11, WiMAX and LPWAN standards [11] and specific routing protocols [13], and cellular mobile network (4G, 5G) low-power wireless access network (LPWAN) IoT standards (LoRa, Sigfox, NB-IoT, etc.) in the wireless segment of the network [14]. The generic architecture of the FiWi access network is shown in Figure 2 [15]. The optical transport network between the optical line terminal (OLT) unit and the optical network unit (ONU) can reach up to 100 km. In the downstream direction, an OLT connects the FiWi access network to the core network, while in the upstream direction it is responsible for scheduling resources toward the ONUs located near mobile users.

The allocation of resources by OLTs is usually dynamic. It involves resource planning in terms of allocating time slots for data transmission to a single ONU device using time-division multiplexing (TDM) or assigning a single wavelength using the wavelength-division multiplexing (WDM) mechanisms. Such dynamic bandwidth allocation (DBA) is a key component of the TDM–PON mechanism that prevents collisions and improves bandwidth utilization through a polling scheme based on determining the ONU bandwidth requirements [16]. Connecting the OLT device with several ONU devices is realized through a shared optical fiber, at the ends of which the passive optical splitters are installed (Figure 2). Such architecture enables point-to-multipoint topology between single ONU and multiple OLT devices.

It is also possible to combine these two solutions using time and wavelength division multiplexing (TWDM), which is characteristic of Next-Generation Passive Optical Network 2 (NG–PON2) and 100G-Ethernet Passive Optical Network (100G-EPON). The TWDM–PON uses the same physical network architecture as TDMA–PON (Figure 2). However, the OLT in TWDM–PON requires multiple transceivers that operate at different wavelengths and the ONUs are equipped with either a tunable transceiver (NG–PON2) or multiple transceivers (100G-EPON) to match specific OLT wavelengths in upstream transmission. The OLT’s transceiver sends packets to all ONUs on the same wavelength in the downstream direction, and each ONU determines whether to receive or ignore the packet that is based on its type and destination address. In order to allow the ONUs to access the shared upstream channel, the OLT employs dynamic wavelength and bandwidth allocation (DWBA) scheduling. Depending on the size of its queue, this system allocates a specific time slot and wavelength for transmission to each ONU.

In classical PON networks, ONU devices terminate at user locations, where it is possible to offer the service to end users exclusively through optical media. However, in FiWi networks, ONU devices are deployed in the characteristic locations (Figure 2). These characteristic locations are planned according to the needs of the spatial coverage of individual BSs or mesh portal points (MPPs) which are integrated with ONU devices. Integrated ONU-BS or ONU-MPP devices represent the interface between the optical and wireless segments of the FiWi access network (Figure 2).

The transmission of optical signals in FiWi access networks can be implemented by RoF and R&F technology. Generally, both RoF and R&F technologies can be deployed in FiWi access networks; however, R&F technology is a very good solution for high-range FiWi networks based on the WLAN standard [14]. In comparison with RoF, the R&F approach also has certain shortcomings in terms of coordination between optical and wireless domains. These shortcomings are related to ensuring QoS and service viability [17]. The R&F concept requires the integration of wireless and optical segments on the physical and media access control (MAC) layers, which then requires the translation of protocols on ONU and BS interfaces. This translation introduces additional cost and complexity in the realization of such networks [17].

The application of FiWi networks is particularly interesting in sensor networks which represent the basis for the realization of IoT concepts in the fields such as telemedicine, smart agriculture, Industry 4.0, smart cities, smart buildings, autonomous driving, etc. This will result in the rapid enlargement of machine-to-machine (M2M) communications, which are characterized by a massive connectivity of communicating nodes (sensors) and transmissions of smaller-size data. Improvements to the FiWi network that could facilitate application within the IoT concept include the use of the millimeter-wave communication band (mmWave) for the transmission at high data rates over shorter distances and the concepts of network function virtualization (NFV) and SDN, which could significantly affect the reduction of capital expenditure (CAPEX) and OPEX of the network [18].

### 2.2. Challenges in Realization of the FiWi Networks

Although the RoF network exploits the advantages of both the optical distribution network and wireless mobile network, the main challenge in the realization of the RoF concept is ensuring appropriate MAC protocol functionality. The merging of wireless and optical network segments has an impact on RoF network functionality, which is primarily reflected in the occurrence of additional propagation delays among different network segments. This can cause the expiration of certain timeouts of wireless MAC protocols that can significantly reduce network performance [14]. For example, when using the IEEE 802.11 standard, a distributed coordination function (DCF) of the MAC technique, the additional propagation delay has a large impact on the performance of the FiWi networks. There are different ways to solve this problem within the RoF concept; however, all of them are solutions that balance the the length of the optical cable and the network bandwidth [14].

One of the possible solutions related to the elimination of the MAC coordination problem is the implementation of R&F technology (Figure 2). While, in the RoF approach, the optical cable is utilized as a medium for transmitting analog signals and management and access control to optical and wireless media is centralized in the central office (CO), in R&F technology, access control and management of optical and wireless media are separated and two different MAC protocols for each used network domain [14]. Therefore, in R&F networks, the optical and wireless networks are combined to create a single, integrated network. In general, R&F networks use distinct MAC protocols in each part of the network and therefore client access control is handled separately [5]. This means that traffic generated solely from wireless communication does not need to be transmitted through the optical network, as in the case of the RoF system. Distributed MAC protocols, such as IEEE 802.11, can avoid the additional propagation delays caused by fiber optic cables, which can negatively impact their performance. This feature allows for greater flexibility in the length of the deployed fiber optic cables and also increases the system’s resilience, since local wireless traffic can still be served even if connectivity with the optical segment is lost [19].

## 3. Energy Efficiency Analyses of Radio-and-Fiber Networks

Although flexible and robust FiWi networks have emerged as an attractive solution for the realization of today’s modern access networks, the low utilization of the optical network elements and high overhead in packet data communication raise an issue of optimizing the EE of FiWi networks. The utilization issue is a consequence of the data traffic variations wherein, during low or no utilization periods, active elements of FiWi networks consume energy as in the periods of moderate or high utilization. Therefore, improving the EE of FiWi networks is a non-negligible challenge and an active research area [6].

Over the years, many power-saving (PS) software techniques have been adopted to increase the EE of hybrid FiWi networks. These mechanisms differ depending on whether the PS technique is implemented in an optical or a wireless domain. Most research studies are focused on increasing the EE of these two domains separately (for the optical back-end and wireless front-end of the network). However, it has been shown that in terms of EE of hybrid FiWi networks, cooperation between PS mechanisms of both domains achieves better results [20]. In this section, the recent optical back-end and wireless front-end PS techniques are first reviewed, and then joint cooperative PS techniques between these two domains are analyzed. Table 1 summarizes related work on the improvement of EE of R&F networks.

### 3.1. Power-Sawing Techniques in the Optical Domain of FiWi Networks

The most popular optical back-end technology currently used in the realization of the FiWi network is the passive optical network (PON). The PON is a cost-effective point-to-multipoint network access architecture. In the PON network, OLT is allocated in the CO and via a passive optical splitter connected with numerous ONUs which are allocated near users (Figure 2) [21]. Due to the constant growth in the number of end users, the largest part of energy consumption in telecommunications falls on access networks [22]. Additionally, in optical access networks, 90% of the energy is consumed for powering ONUs and, therefore, the energy-conservation techniques for ONU devices are of great importance [23]. Moreover, in the PONs, the OLT is continuously occupied with transmitting and receiving data, making it unsuitable for energy-saving strategies. On the other hand, the ONU’s transmitter is idle for the majority of the time and it is appropriate for the implementation of energy-saving strategies [16].

**Figure 3:**
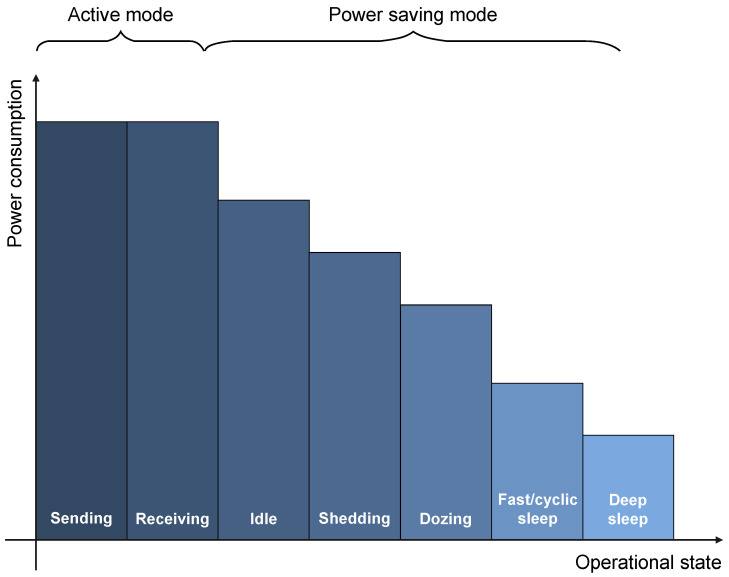
ONU power consumption under different active and energy-saving states.

Reducing ONU device power consumption can be achieved primarily through different PS operation modes prescribed by the ITU-T standard [24] (Figure 3). These PS modes of operation are characterized according to the operational state of the ONU receiver and transmitter. Accordingly, the ONU operation state can be in one of the following energy-saving modes: ONU power shedding, ONU dozing or ONU (fast and deep) sleeping modes (Figure 3). The power-shedding mode conserves energy by switching off only unnecessary ONU features, while keeping the receiver and transmitter fully operational. Thus, in the power-shedding mode, minimal power savings are ensured (Figure 3), while the best possible performance is maintained.

**Table 1:** Summary of related works on PS methods in R&F networks.

	Reference	PS Method	Summary of Contributions Related to the Improvement of FiWi Network Energy Efficiency
ONU sleeping mechanisms	[25]	ONU sleep mode with ALR	Hybrid PS technique that includes adaptive link rate control and sleep functions.
	[26]	WSM	A PS mode that combines the doze and cyclic sleep modes into a single mode.
	[27]	ONU sleep mode and WSM	Comparative performance analysis of fast/cyclic and watchful sleep modes
	[28]	WSM operation mode with DBA	Performance of watchful sleep mode that utilizes the dynamic bandwidth allocation.
	[29]	AWSM for UNUs	An introduction of adaptive watchful sleep mode
	[30]	Load-adaptive ONU sleeping scheme	Load-adaptive ONU PS mechanism that adjusts the number of sleeping ONUs based on the overall load on the network.
	[16]	PS mechanism based on OSC, GDBA and TSC components	A PS method based on the SIEPON standard.
	[31]	Decentralized PS mechanism based on ONU queue manager, TRx controller, sleep manager, OLT queue manager and GDBA components	Decentralized PS solution based on the SIEPON standard.
	[32]	Optimization of sleep interval using ANN	Determination of optimal fast/cyclic sleep interval for energy-efficient XG-PON. The ANN model is used to estimate the optimal sleep interval values.
Wireless power-saving techniques	[33,34]	Integer linear programming (ILP) optimization model and heuristic algorithm	An approach for finding the most efficient way to save energy in wireless access networks using heuristics.
	[35]	Transmit power scaling and on/off switching	Extensive studies on the impact of changing the transmit power and turning the BSs on and off on the instantaneous power consumption of macro BSs. Real-world measurements are used from a range of different macro BSs to develop linear power consumption models.
	[36]	Adaptive PSM	An adaptive PS method in wireless networks that are based on the IEEE 802.11 standard.
	[37]	Scheduled PSM based on a time-slicing mechanism	A PS method based on a time-slicing mechanism in a multi-traffic environment with high background traffic.
	[38]	PSM based on the execution of the heuristic algorithm	A generic power management model according to which the wake-up scheduling mechanism is controlled by the AP. Proposes two heuristic algorithms to address the downlink scheduling optimization problem, identifying the importance of tuning the length of the beacon interval in order to conserve energy and reduce delay.
	[39]	C-PSM	A centralized PS mechanism that improves the EE of wireless clients in an 802.11 infrastructure network.
	[40]	SAPSM	A PS method that uses a ML classifier to assign priorities to applications, where applications classified as high-priority can switch to active mode, while traffic classified as low-priority is optimized for EE.
	[41]	A ML method of identifying and categorizing network traffic	A ML-based approach for optimizing power saving in WLANs by classifying network traffic based on contextual factors, and adjusting the listen interval accordingly.
	[42]	Overview of PS methods	Overview of the power supply system parameters for powering the BS sites with renewable energy sources. Approaches for reducing telecom operator energy and CAPEX based on different air-conditioning systems for BS sites.
	[43]	Overview of PS methods	Overview of the renewable energy sources for powering base station sites. Comparison of the EE among hybrid systems that use multiple renewable energy sources and systems that use a single renewable energy source.
	[44]	Save energy and maximize connectivity (SEMC) algorithm	A generic algorithm for ad-hoc wireless networks that conserves energy and maintains good connectivity through adjusting transmission range and choosing a transmission time based on data rates, which results in reduced transmission power and energy savings.
Cooperating optical and wireless techniques	[21]	ONU sleep mode with PSM	A method for determining the optimal sleep period and behavior for optical ONUs for improving throughput and reducing energy consumption.
	[45]	ONU sleep mode with radio interface standby	A wireless–optical topology reconfiguration scheme that enables integrated energy saving through reconfiguration of the optical topology using ONU sleep mode and the wireless topology using radio interface standby.
	[46]	ONU sleep mode with PSM and adaptive PSM	A method for controlling the ONU sleep period based on the energy control mechanism of wireless stations.
	[47]	ONU sleep mode with powering off radio interfaces	An ONU sleep algorithm for dynamic scheduling of the power states of ONUs based on their traffic profile and load thresholds. An algorithm for dynamic radios turning off in order to reconfigure the wireless topology by dynamically controlling the power states of radios.
	[6]	ONU sleep with adaptive frame aggregation and load transfer mechanism	Proposed adaptive frame aggregation mechanism that optimizes energy consumption by adjusting frame lengths based on channel quality. Proposed the delay-aware load transfer mechanism that maximizes ONU sleep time and ensures reliable service transmission by allocating traffic load based on QoS requirements.
	[48]	ONU sleep mode with PSM and DBA	A PS scheme that coordinates power-saving modes for wireless stations, APs and ONUs, in order to reduce energy consumption.
	[20]	TDMA mechanism between ONU and wireless station and between OLT and ONU with DBA	A technique that aims to reduce delays and improve EE by organizing the system into clusters of ONUs and using an equal partition approach. Using this approach the ONUs in the back-end and the wireless stations in the front-end are active only during certain timeslots in the TDMA cycle.
	[49]	Load transfer region sleep mechanism between ONU and wireless stations	A collaborative sleep mechanism that uses load transfer to determine which nodes should sleep and adjusts routes for affected services based on service priority.
	[50]	Genetic algorithm, teaching-learning-based optimization, spiral update positioning and encircling prey mechanism	Several different ONU placement optimization algorithms are compared in extensive simulations.

In ONU dozing mode, the ONU transmitter is powered off for a considerable amount of time, while the ONU receiver maintains an active state at all times. This corresponds to even lower instantaneous power consumption of the ONU dozing mode when compared with the power consumption of the power-shedding mode (Figure 3). The ONU sleeping modes are, according to [24], further divided into two subgroups: deep sleep and fast sleep (also known as cyclic sleep) (Figure 3). The ONU sleeping modes are characterized by powering off both the ONU receiver and the transmitter for a considerable amount of time. When using ONU sleeping mechanisms, it is necessary to make a trade-off between energy savings and network performance. Due to powering off the receiver and the transmitter during the entire PS mode period, during the ONU deep sleep mode maximum energy conservation can be achieved at the cost of significant performance degradation. On the other hand, during the ONU fast/cyclic sleep mode, the transmitter and the receiver alternate between on and off periods, forming sleep cycles, which results in lower energy savings when compared with the energy savings of deep sleep mode (Figure 3).

Another PS method in PONs is the implementation of the adaptive link rate (ALR) concept. It is based on energy reduction that can be achieved by adapting different transmission rates between optical devices [51]. In optical access networks where multiple data transmission rates are available (e.g., GPON and G-EPON), reducing transmission rates in low-traffic periods can contribute to the reduction of the energy consumption of the optical unit and this reduction improves network EE. Moreover, these PS techniques can be combined with ONU sleep modes for even greater energy savings. The authors in ref. [25] proposed a hybrid scheme that combines the ONU sleep mode with ALR mechanisms in order to improve the EE of 10G-EPON systems (Table 1). Such a hybrid PS scheme activates the ONU sleep mode in the absence of network traffic, while in the presence of network traffic, the ALR function for adapting the downlink data rates is activated based on the levels of traffic load. It was shown in ref. [25] that such a hybrid PS approach can contribute to significant energy consumption reductions in the optical part of the network.

In addition to the aforementioned PS modes, a newer PS scheme called watchful sleep mode (WSM) has emerged and is also included in all major PON standards [52,53,54]. The WSM acts as a unified solution that combines cyclic sleep and doze PS modes. It eliminates the need for mode negotiation between optical units and maximizes the ONU’s EE. It is expected that WSM will be used as the only PS mode for PONs in future (Table 1) [26]. In ref. [27], the authors conducted a performance comparison between PS fast/cyclic sleep modes and a WSM for a GPON ONU and concluded that both approaches offer similar energy savings. However, the WSM performed better regarding state transition latency (Table 1). The simulation results in ref. [28] showed a decrease in downstream and upstream transmission delays with a significant energy savings when WSM was implemented in combination with dynamic bandwidth allocation (DBA) in a 10-gigabit-capable passive optical network (XG–PON) (Table 1). In addition, the authors in ref. [29] recently proposed a new energy-efficient scheme called adaptive watchful sleep mode (AWSM). The AWSM increases the energy savings of the standard WSM scheme by minimizing the ONU receiver’s active (on) time during the watch state (Table 1). In ref. [30], a PS strategy was proposed for edge-enhanced metro FiWi networks using matching game theory. The proposed method optimizes the number of sleeping ONUs depending on network traffic (Table 1) and thus improves network EE.

Furthermore, the IEEE service interoperability in the Ethernet passive optical networks (SIEPON) work group (SIEPON 1904.1) standardized two additional PS mechanisms, namely, transmitter (Tx) and transceiver (TRx) sleep modes for EPON systems [55]. Similar to the doze PS mode of the ITU-T standard, in the Tx sleep mode, ONU transmitter subsystems can enter the sleep (PS) mode, while the receiver components remain fully operational. In contrast, the TRx sleep mode is equivalent to the ITU-T fast/cyclic sleep mode and this PS approach enables entering the sleep (PS) state for both the transmitter and receiver subsystem of the ONU. Compared with the Tx sleep mode, the TRx sleep mode is more energy efficient at the expense of the increased delay. In both the Tx and the TRx sleep modes, the OLT is responsible for controlling the ONU sleep intervals, with the main task of determining the right sleep interval that can satisfy both the delay and the energy-saving requirements [56].

To optimize Tx sleep interval wile satisfying QoS in TDM PON systems, the authors in ref. [16] proposed a SIPEON-based energy-saving scheme (Table 1). The proposed scheme is based on adding new components to the conventional PON architecture, namely the ONU sleep controller (OSC), the green DBA (GDBA) mechanism, and the Tx sleep controller (TSC). The OSC and GDBA components are a part of the OLT hardware architecture and they are used to calculate the ONU Tx sleep periods based on received report messages. The TSC component, as part of the ONU, is responsible for ONU transmitter control and for monitoring the incoming traffic. The TSC enables the exit and entering of ONU in sleep mode according to instantaneous traffic intensity. The simulation results presented in ref. [16] showed that this energy-saving scheme can decrease the Tx power consumption of ONU while satisfying desired SIEPON and ITU-T QoS performance requirements.

In contrast to the centralized sleep mechanism introduced in the SIPEON standard, the authors in ref. [31] proposed a decentralized energy-saving solution that is also based on the SIPEON standard (Table 1). In the PS solution proposed in ref. [31], instead of the OLT unit, the Tx or TRx sleep mode is initiated by the ONU. To achieve this, the ONU hardware architecture is enriched with new components known as the ONU queue manager and TRx controller, while the OLT hardware architecture is enriched with the sleep manager, OLT queue manager and GDBA mechanism. The ONU Tx sleep duration is calculated by the TRx controller and reported to the OLT. Then the sleep manager calculates the sleep intervals of the ONU receiver and, based on these intervals, the type of sleep mode is determined. The obtained simulation results showed that significant energy consumption reductions could be achieved in the optical segment of the network while satisfying QoS requirements.

In the past, the fast/cyclic sleep mechanism of the ONU has been extensively researched to reduce energy consumption in XG-PON. However, due to the emergence of new types of network traffic with stringent demands, further improvements in sleep time interval selection are required. Hence, the paper [32] proposed the use of an artificial neural network (ANN) to enable the ONU to determine the optimal sleep time interval values by learning from past experiences. The M/G/1 queueing system was used for theoretical analysis prior to simulation, and the ANN was trained and tested for the XG-PON network to make optimal sleep time interval decisions. The results indicate that as the network load increases, sleep time interval decreases for both methods. The ANN network records a wider range of sleep time interval values than the theoretical values. As a result, these findings will enable network operators to determine the optimal sleep time interval values at the current network conditions with more flexibility.

### 3.2. Power Saving Techniques in the Wireless Domain of FiWi Networks

Besides the optical domain, different PS techniques have been developed in the wireless domain of FiWi networks. Since the highest consumers of energy in the radio part of the network are wireless network (front-end) devices (e.g., BSs, APs, LPWAN gateways, etc.), different solutions have been proposed for improving the EE of the wireless segment of FiWi networks. Most of the proposed solutions are based on dynamic control of the activity state of wireless network devices in accordance with traffic variations. Besides ONTs, wireless network devices can also be, at a certain moment, in an active or in a sleep state. Whether it is transmitting or being idle in the active state, wireless network devices consume significantly more energy compared with the sleep (partially or fully turn-off) state [57]. Hence, different PS approaches have been presented in refs. [33,34,35,58] as techniques for wireless network station on- and off-activity adjustments depending on the data load.

Moreover, a mechanism called an adaptive power saving mechanism (PSM) was introduced in ref. [36], which, instead of inefficient fixed wake-up intervals, uses an adjustment constant in order to adjust the wake-up intervals of wireless network devices in a more efficient manner (Table 1). Similar to ONU sleep techniques, using PSM and adaptive PSM, the wireless network station needs to buffer the incoming data, which consequently causes additional transmission delay [18].

To address these issues associated with initial PSM, the authors in ref. [37] presented a scheduled access point (AP)-centric PSM protocol based on a time-slicing mechanism. The proposed protocol enables the improvement of energy-efficient scheduling of AP activity at the expense of minimal delay in a multi-traffic environment with heavy background traffic (Table 1). The authors in ref. [38] developed a generic power management model (GPMM), according to which the wake-up scheduling mechanism is controlled by the AP. Additionally, to address the downlink scheduling optimization problem and to achieve more energy savings, in ref. [38] two heuristic algorithms for optimal AP activity adjustments were proposed (Table 1). Furthermore, the authors identified the importance of tuning the length of the beacon interval in order to conserve energy and reduce delay. Since both of the aforementioned scheduling techniques are computationally demanding, the authors in ref. [39] proposed a centralized power-saving mode (C-PSM). In order to reduce latency and increase EE, the C-PSM approach uses traffic pattern statistics to calculate the optimal AP listening intervals, beacon intervals and congestion window size (Table 1).

Effective management of WLAN power consumption on smartphones can have a significant impact on energy consumption. The authors in ref. [40] have shown through experiments that the WLAN power management process on various smartphones is autonomous and occurs entirely at the driver level. However, the limitation of driver-level implementations is that essential power management decisions can only be made by monitoring packets at the MAC layer, which disables distinguishing between applications. As a result, each application has an equal chance to consume more energy and determining which applications can impact WLAN power management is crucial. To solve this problem, the authors have introduced a smart adaptive power save mode (SAPSM). SAPSM uses a machine learning (ML) classifier to assign a priority label to each application. Only applications that have high-priority can affect the client’s behavior to switch to active mode, while traffic with low-priority is optimized for EE. It is shown that the SAPSM implementation on an Android smartphone device significantly improves EE under typical usage scenarios.

As an extension of the SAPSM approach, the authors in ref. [41] have proposed a new classification method of network traffic using ML classifiers to optimize WLAN power saving. The approach utilizes the contextual degrees of traffic interaction in the background for ML classifier applications. The output traffic is then classified to optimize context-aware listen interval PSMs. The study evaluates the performance of several ML classifiers using a real-world dataset of several smartphone applications that enable the reflection of various types of network interactions and behaviors.

In addition to the aforementioned PS methods in the wireless domain of the FiWi network, methods for powering the locations of wireless network devices (AP and BS) using renewable energy sources have also been proposed [42,43]. These methods represent an effective way to save energy and reduce OPEX for network owners [42]. Due to the lack of ability for BSs to access the electricity grid, having limited access to daily power supply or simply having large expenses for consumed electricity, mobile network operators have a significant interest in using renewable energy sources for powering remote BS sites. Therefore, using renewable energy sources can reduce OPEX and improve the energy efficiency of the wireless domain in FiWi networks.

Furthermore, the authors in ref. [44] proposed an algorithm that aims to conserve energy and maintain connectivity in mobile ad-hoc networks (MANETs). The algorithm aims to conserve energy in nodes that have limited battery life and maintain connectivity between nodes, which is crucial for route discovery. The proposed algorithm is a generic algorithm that can be applied in various situations and operates at layer 2 of the Open Systems Interconnection (OSI) model, making it independent of routing algorithms. The simulation results showed that the proposed algorithm significantly reduces energy consumption while maintaining good connectivity over time.

### 3.3. Cooperating Power-Saving Techniques in the Optical and Wireless Domain of FiWi Networks

Since the FiWi network consists of optical and wireless domains that are realized with two different technologies with versatile PS mechanisms, cooperation between these two PS mechanisms is found to be a desirable option. It was shown in refs. [21,46] that unsynchronized PS mechanisms between optical and wireless network domains lead to the degradation of the performance and EE of the FiWi network. To reduce such degradations, the authors in ref. [21] proposed a method that increases ONU EE and reduces wireless network stations’ latency without sacrificing throughput in a FiWi network. The proposed cooperative PS method simultaneously executes the ONU sleep mechanism in the optical domain and the PSM mechanism in the wireless domain of the network (Table 1).

For the realization of joint EE improvement in both domains of the FiWi access network, another interactive PS method based on the wireless–optical topology reconfiguration (WOTR) technique is proposed in ref. [45] (Table 1). The proposed reconfiguration scheme uses the ONU sleep method in combination with the method which puts the radio interface (RI) in the standby state. Implementation of this method is realized through two interactive modules: one for the optical back-end and one for the wireless front-end. Through simulations, the authors in ref. [45] demonstrated significant energy consumption reduction with negligible network throughput degradation when compared with optical-only PS schemes.

Due to the issues related to the increased latency and degraded EE caused by the simultaneous use of unsynchronized PS schemes in the optical and wireless domains of the FiWi network, the authors in ref. [46] proposed a cooperative ONU sleep scheme that dynamically adjusts the ONU sleep period according to the conditions in the wireless domain (Table 1). By proposing an integrated EE scheme that jointly schedules both ONU and radio sleep states, the authors in ref. [47] designed an energy-efficient FiWi network in which power consumption optimization was based on the developed heuristic algorithms (Table 1). The proposed network design uses an ONU sleep mechanism that dynamically schedules ONU sleep periods and a “Radios Off” algorithm for wireless topology reconfiguration. The QoS is ensured by employing wireless rerouting. Moreover, the frame aggregation scheme introduced in WLANs for boosting the overall performance of the wireless front-end was proven in ref. [59] to be an effective approach for improving the EE of the FiWi networks. In order to reduce the power consumption of the FiWi networks, authors in ref. [6] present an adaptive frame aggregation and load-transfer scheme. The proposed scheme jointly maximizes the EE and ONU sleeping periods of the FiWi IoT networks, realized as PON in the optical domain and wireless mesh network (WMN) in the wireless domain (Table 1).

The authors in ref. [48] proposed a cooperative PS scheme called the energy-conservation scheme for FiWi networks (ECO-FiWi) that synchronously deploys wireless front-end and optical back-end PS techniques and integrates them into a dynamic bandwidth allocation (DBA) procedure by leveraging the time division multiple-access (TDMA) operations (Table 1).

**Table 2:** Summary of the related works on PS methods in RoF networks.

References	PS Method	Summary of Contributions Related to the Improvement of FiWi Network Energy Efficiency
[60]	Number and directivity of antennas and antenna position optimization	Method for improving EE of a DAS by increasing the number of antennas and optimizing the antenna position.
[61]	Antenna unit output power optimization	Comparison of the EE of optimized narrowband single-service and broadband multi-service DAS solutions.
[62]	Antenna position optimization and the selection of the optimal number of antennas	Investigation of the EE of the RoF DAS technologies by measuring the power consumption of 802.11 APs and smartphones. Proved the existence of the optimal number of distributed antennas for a given indoor environment topology.
[63]	Selection of the optimal number of antennas with data frame aggregation mechanisms	A method for evaluating and optimizing energy consumption in 802.11n RoF DAS systems. Proved the existence of an optimal number of distributed antennas for a given scenario, and that the data frame aggregation mechanisms can further improve EE.
[64]	Centralized RoF architectures using dedicated RoF links for each cell	An EE model for RoF networks confirmed better network EE when designed with small cells and when the energy usage of the remote units surpasses a certain threshold.
[65]	Analyses of the impact of E/O/E conversion, number of services and wireless network capacity on EE of the RoF links	Confirmed that E/O/E losses degrade EE on an optical link using the A–RoF technique. Confirmed that the D–RoF link shows the degradation of EE at higher Nyquist zones due to RF signal reconstruction. Confirmed that the wireless bandwidth can improve the EE of both, the A–RoF and D–RoF connections and that the amount of energy savings in the presence of multiple services depends on the specific wireless environment.

The study found that the use of TDMA in FiWi networks significantly improves the energy efficiency while maintaining delay performance, with the feature of increasing energy savings proportionally with traffic load. Another cooperative mechanism called the delay-controlled and energy-efficient clustered (DEC) TDMA scheme was presented for the FiWi network in ref. [20] (Table 1). This is a cooperative scheme that jointly considers the EE of the OLT, ONU and wireless network devices by leveraging the TDMA technique. Moreover, to further decrease delay and energy consumption, the DBA mechanism was used to allocate data transmission slots between wireless network devices and ONU, and between ONU and OLT. It was shown that the EE of the DEC TDMA FiWi network was equivalent to ECO-FiWi, but in terms of transmission delay, DEC TDMA outperformed the ECO-FiWi scheme.

In order to achieve energy conservation and ensure satisfactory QoS, a collaborative sleep technique between optical and wireless nodes was developed in ref. [49] (Table 1). This technique consists of two sub-techniques. The first one is a technique based on load balancing for achieving optimal planning of transmission route selection, and the second one is a cooperative sleep technique based on load transfer using priority-based rerouting. The simulation results showed that this technique can achieve high throughput and EE with low latency for high-priority services. In ref. [50], the authors proposed a framework that simultaneously focuses on ensuring the EE and survivability of the FiWi networks using optimization algorithms for ONU placement (Table 1). Several different optimization algorithms were compared in extensive simulations, namely, genetic algorithm, teaching–learning-based optimization, spiral update positioning and encircling prey mechanism. The authors concluded that the genetic algorithm provides the best performance regarding EE, with an average performance in terms of the FiWi network survivability.

## 4. Energy Efficiency Analyses of Radio-over-Fiber Networks

**Figure 4:**
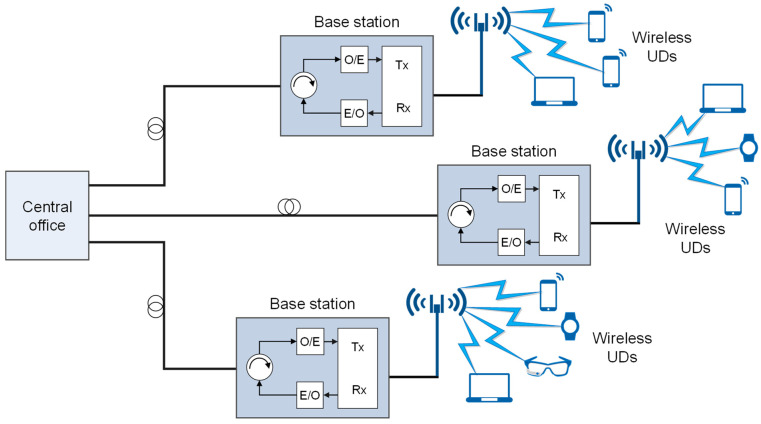
Generic RoF architecture.

The implementation of the FiWi networks using the RoF technology is based on exploiting optical fiber as a means for the direct transport of radio frequency (RF) signals among one or more remote access (antenna) units (RAU) and network operator central office (CO) (Figure 4). The CO is responsible for managing access to both the optical and wireless domains [18].

The optical modulation of the RF signal is realized through electrical-to-optical (E/O) conversion, which is required for transmitting an RF signal over an optical–fiber link. Compared with traditional coaxial-based RAN systems, the RoF network paradigm offers several advantages. Those advantages are related to the high bandwidth of fiber links with high data rates and low signal attenuation. This allows greater transport distances and better resistance to electromagnetic noise and thereby increases the flexibility of the allocation of network devices, reduces BS size due to the BS design with RRUs and BBUs, increases installation flexibility, allowing multiple operators to share the same RoF network and improves the EE through dynamic resource allocation realized using the centralization of baseband functions at CO premises [66].

**Figure 5:**
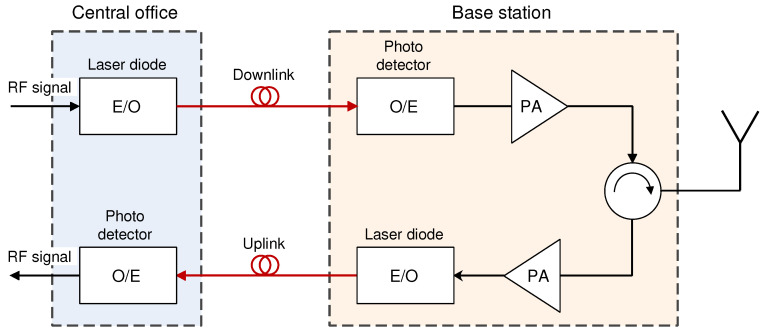
General A–RoF optical link architecture.

**Figure 6:**
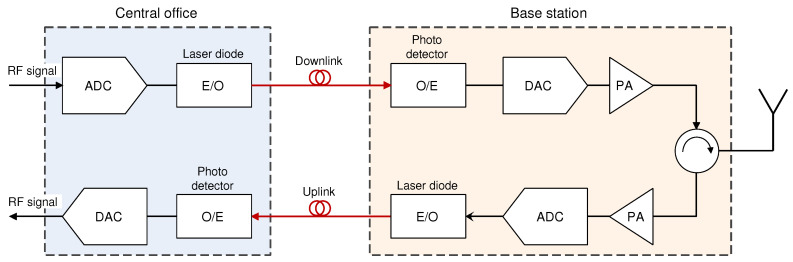
General D–RoF optical link architecture.

Depending on how the RF signal is transmitted through an optical–fiber link, there are two basic RoF communication techniques known as digitized RoF (D–RoF) and analog RoF (A–RoF) [67]. In the A–RoF system (Figure 5), the transmission of the RF signal is carried in the analog domain, where analog RF signal is used for light modulation of the optical signal transmitted in the fiber. On the other hand, in the D–RoF system (Figure 6), the D–RoF signal is digitized prior to transmission over an optical communication link.

For example, the MFH link between the RRU and the BBU designed for the LTE advanced (LTE-A) C-RAN architecture based on a common public radio interface (CPRI protocol is a prominent representative of the D–RoF. The main concept of such an MFH solution based on the CPRI interface is to use a digitized version of the baseband signal before being transmitted over the optical–fiber link. Although the CPRI-based fronthauling solution has proven to be effective for previous mobile network generations (i.e., 2G, 3G and 4G), the D–RoF approach has one major issue. Namely, the CPRI has shown to be bandwidth-inefficient, as it uses a constant data rate for transmitting the signals [68]. Hence, this bandwidth inefficiency can become critical in 5G networks using carrier aggregation (CA) and mMIMO techniques wherein high bandwidth and capacity requirements are expected. Moreover, the CPRI does not support flexible rerouting that allows automatic RRU switching to another BBU and the data rate is dependent on a number of antennas which can also be critical in 5G mMIMO implementations [69].

Presumably, the D–RoF-based solutions may have been sufficient for the early stages of the 5G network, but as 5G reaches its full potential, digitized optical MFH transmission based on the conventional CPRI protocol will generally not be sufficient [70]. Analog–optical transmission, commonly referred to as the A–RoF, presents a traditional RoF technique that has promising potential to overcome D–RoF’s CPRI limitations (Figure 5). Analog modulation of the optical signal with no prior digitization addresses bandwidth limitations, making it more suitable for high-bandwidth networks. On the other hand, due to the characteristics of the fiber link, transmitted A–RoF signals can experience numerous link impairments such as attenuation, chromatic dispersion and fiber nonlinearities [71]. Depending on the carrier frequency used for modulation of the optical signal, two common A–RoF techniques are usually considered, i.e., the radio frequency-over-fiber (RFoF) and the intermediate frequency-over-fiber (IFoF). The main difference between the RFoF and the IFoF techniques is in the carrier frequency used for modulation of the optical signal for transmission over a fiber–optical link. In the case of the RFoF technique, the analog signal directly modulates the optical signal, while in the IFoF technique, the signal of intermediate frequency is used for optical signal modulation.

### 4.1. Approaches for Improving Energy Efficiency in General RoF Networks

**Figure 7:**
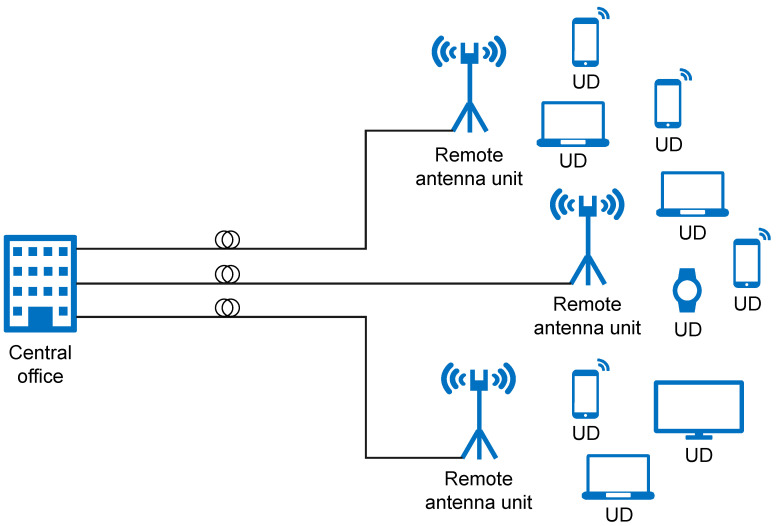
RoF DAS architecture.

As the RoF integration and mobile broadband access techniques have been developed, studies focused on improving the EE of the RoF networks have been performed. An overview of research activities related to the EE improvements of the ROF-based systems is presented in Table 2. In ref. [60], it was shown that RoF-distributed antenna systems (DAS) are the most appropriate architecture for the deployment of high-capacity wireless communication systems (Figure 7). The authors in ref. [60] demonstrated that, for an active DAS system that utilizes optical fibers, there is a specific number of antennas that results in the highest EE when the power consumption of both the radio and optical components is considered (Table 2). In contrast to traditional WLANs, the RoF DAS architecture uses multiple remote antenna units instead of individual APs while maintaining all signal processing functions at a CO (Figure 7). This allows for a reduction in the complexity and power consumption of the remote antenna units [63].

One way to optimize the RoF DAS performance is to minimize the power consumed at each remote antenna unit, while providing wireless coverage to a specific area. In ref. [61], the authors proposed a solution to decrease energy usage in the RoF DASs by adjusting the RF output power at the antenna units for optimal efficiency (Table 2). They found that a narrow-band DAS is more energy efficient than a wide-band DAS, due to the improved efficiency of the power amplifiers, for a single wireless service. However, when more than two services are needed, a multi-service broadband DAS can be more energy efficient than multiple narrow-band DASs when used for offering individual wireless services.

**Figure 8:**
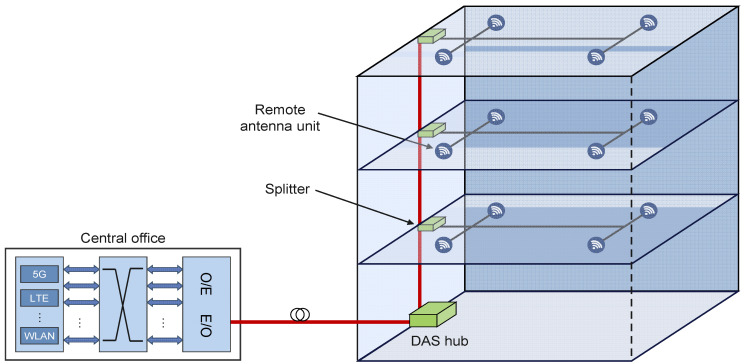
An example of indoor DAS implementation.

It was shown in ref. [62] that DAS architecture can enhance the coverage and performance of wireless communications within indoor RoF DAS deployments (Figure 8). In a specific study, the authors suggested a method to determine the location of distributed antennas to optimize network capacity based on the probability of non-uniform user presence (Table 2). They also used power consumption measurements of smartphones and 802.11 APs to calculate the EE of a DAS that uses radio-over-fiber (RoF) technology. For the given indoor environment topology, the simulation results indicated that there was a specific number of distributed antennas that maximized the EE of the system.

In ref. [63], the authors studied the EE of IEEE 802.11n-based RoF DAS architectures (Figure 7) and developed a method for evaluating and optimizing the energy consumption in these systems (Table 2). They found that there was an optimal number of distributed antennas for specific AP implementation scenarios, and that the aggregation mechanisms of the IEEE 802.11n standard could further improve the EE in the RoF DAS. Additionally, they demonstrated that MAC protocol data-unit-aggregation techniques are more effective in providing higher end-to-end throughput and greater EE than MAC service data-unit-aggregation schemes in the IEEE 802.11n RoF DAS.

The authors in ref. [64] conducted a study on the EE of indoor networks, which provide high-speed mobile access to end users using hybrid RoF technology (Table 2). Using a validated EE model, they found that while individual RoF links may not be as energy efficient as traditional baseband-over-fiber links, the RoF networks could be more energy efficient when carefully designed with small cells and when the energy usage of the remote units is above a certain level.

In ref. [65], for an indoor network, a theoretical evaluation model was presented in order to evaluate the effect of wireless bandwidth, multiple services and loss due to electrical–optical–electrical (E/O/E) conversion on the EE of the optical links in A–RoF- and D–RoF-based networks (Table 2). It was shown that E/O/E loss had a large impact on the EE of the optical link when the A–RoF transmission technique was used. On the other hand, it was shown that the D–RoF link was less susceptible to E/O/E loss. However, the D–RoF link showed a degradation of the EE due to RF signal reconstruction at higher Nyquist zones. Furthermore, it was shown that the EE could be improved on both the A–RoF and the D–RoF connections by increasing the wireless bandwidth. It was also shown that in the presence of multiple services, additional energy savings depend on the wireless environment.

### 4.2. Approaches for Improving the Energy Efficiency of Cloud Radio Access Networks

#### 4.2.1. Energy-Saving Potential of Cloud Radio Access Network Architecture

The usage of optical fiber instead of coaxial cabling for connecting BBU and RRU locations in the D-RAN network architecture constitutes the basis for the realization of next-generation cellular mobile networks (Figure 1) [72]. Due to communication between RRUs and BBUs realized over fiber links, such systems can achieve communication over longer distances than traditional BSs, having collocated RRU and BBU in the same cabinet or having the connection between RRU and BBU using coaxial cables or waveguides at the BS site (Figure 1).

**Figure 9:**
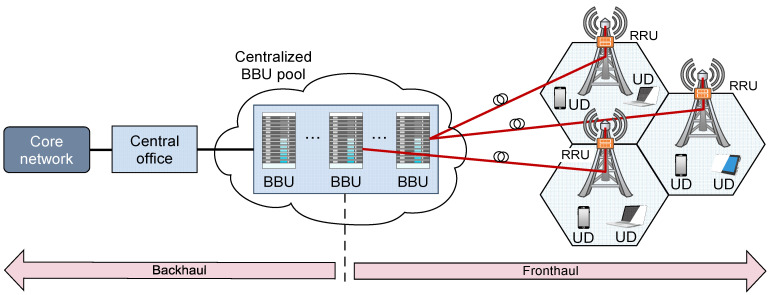
C-RAN architecture.

The D-RAN concept is particularly suitable for the realization of RAN as a HetNet with the large number of distributed small cells realized as elements of DAS. Small-cell communication systems realized through DAS show potential for increasing the capacity and data rate of RANs. However, such RAN communication systems must be simple and have low power consumption. To meet these requirements, the RAN architecture is evolved from D-RAN to cloud-based RAN (C-RAN). The C-RAN architecture involves separating the BBU of traditional cell sites from remote RRUs and allocating the BBUs in a central cloud location (Figure 9). This allows complex and power-inefficient traditional macro BS sites to be simplified. Simplification means the allocation of just RRUs at the BS site (Figure 9). This approach reduces BS site power consumption due to RRU natural air conditioning and decreases BS site maintenance costs due to the lower amount of components installed at the BS site [73].

Although three communication standards in D-RAN systems have been developed (i.e., Open Base Station Architecture Initiative (OBSAI), CPRI and Open Radio equipment Interface (ORI)) for digital–optical interface communication between RRU and BBU [74], the CPRI has become the most common D–RoF protocol, which is also predominantly used in C-RAN networks (4G/5G C-RAN). It defines ten different options in terms of transmission bit rates, which range from 0.61 Gbit/s for Option 1 to 24.33 Gbit/s for Option 10 [75].

**Table 3:** Summary of the related works on PS methods in C-RAN networks.

References	PS Method	Summary of Contributions Related to the Improvement of FiWi Network Energy Efficiency
[76]	CnR algorithm	A method for estimating the resource utilization rate of BBUs. Additionally, the CnR algorithm to save energy in the BBU pool is presented, and it is shown that the proposed algorithm is effective at decreasing energy consumption in the BBU pool and overall system.
[77]	Wake-on-RRU and wake-on-BBU approach	An approach that uses WoL packets sent by the RRU to wake up BBUs and an approach that uses WoL packets to wake up BBUs sent by the controller in the BBU pool.
[78]	Dynamic resource provisioning (DRP) algorithm	A dynamic resource-allocation algorithm to select active RAUs and consolidate virtual machines onto computing units in order to minimize energy consumption in C-RANs. In order to achieve this goal, the proposed algorithm uses a context-aware scheme to minimize the number of virtual machine migrations.
[79]	Graph partitioning algorithm and rejoining algorithm	A scheme for associating BBUs and RRUs based on graph partitioning and rejoining in order to minimize power consumption.
[80]	Power control algorithm	A power control algorithm based on mobility prediction for improving the EE of 5G H-CRAN.
[81]	H-CRAN energy-efficient radio resource management (HERM) algorithm	The HERM algorithm to solve the network EE optimization problem. The results showed that the developed algorithm significantly improves the EE of H-CRAN.
[82]	MIMO–RoF system	An adaptive RoF system for next-generation C-RANs that takes into account energy consumption, capacity per wavelength and distribution range.
[83]	Particle-swarm optimization (PSO), quantum PSO (QPSO) and genetic algorithm (GA) approaches	The optimal number of virtual machines that maximize the EE of C-RAN.
[84]	Heuristic-Assisted Deep Reinforcement Learning (HA-DRL) BBU aggregation scheme	An aggregation scheme for BBU based on HA-DRL that ensures both energy efficiency and guaranteed QoS.
[85]	Double Deep Q Network (DDQN) resource allocation framework	Framework based on DDQN resource allocation method that maximizes the overall EE in C-RAN.

The C-RAN architecture (Figure 9) was introduced in 2010 by China mobile as a centralized solution to better support the requirements of future HetNets [86]. Since traditional RAN systems suffered from numerous challenges related to increased CAPEX and OPEX with spectral and energy inefficiency, in the cloud centralized solution has become a necessity in designing future RAN systems for next-generation mobile networks. The C-RAN thus presents a logical evolution step from the D-RAN (and the centralized D-RAN), where baseband processing is separated from the cell site and is performed in the remote CO (Figure 9). In comparison with the centralized D-RAN (Figure 1), the C-RAN architecture enables complete RAN architecture to be based on the cloud-centralized management and control paradigm (Figure 9). In remote CO, multiple BBUs can be aggregated in a BBU pool that can utilize the computational resources of multiple BSs (Figure 9). The RRUs are connected to the BBU pool through C-RAN mobile fronthaul (MFH) realized with high-speed and low-latency optical links [87]. The distance between RRUs and the BBUs physically located in the cloud can be up to hundreds of kilometers away. With such an approach, centralized operation and management of overall RAN can be ensured [88]. This BBU centralization can significantly reduce mobile operator OPEX, since multiple BBUs aggregated in the same central equipment room (CO) in the cloud can share the same resources (e.g., power supply, air-conditioning) and thus improve the EE of the network [89].

Furthermore, in C-RAN network architecture, RRU design and functionality are much simpler, and this further contributes to the reduction of BS site power consumption and maintenance costs. Additionally, the C-RAN has an architecture suitable for the implementation of cooperative techniques such as coordinated multi-point (CoMP) processing technology. The cooperation of multiple BBUs in the large cloud BBU pool using CoMP technology enables the sharing of different system information in the cloud. This can improve the spectral and EE of the RAN and also can contribute to alleviating inter-cell interference (ICI) of the densely deployed small cells [86].

Therefore, through the implementation of the cloud-computing paradigm, the C-RAN architecture is able to aggregate multiple BS resources into a central BBU pool in the cloud. According to ref. [90], with such centralized architecture, it is possible to optimize multi-cell cooperation processing, which results in more energy-efficient operation of RAN than those having decentralized BBUs in the cloud. Therefore, full implementation of the C-RAN architecture can bring considerable potential energy savings to mobile network operators in the future.

#### 4.2.2. Techniques for Improving Energy Efficiency in Cloud Radio Access Networks

Since energy efficiency has become one of the main concerns when designing RAN, some initial research analyses have been published with respect to improving the EE of C-RAN networks and they are summarized in Table 3. In the case of the latest 5G mobile cellular networks, the heterogeneous C-RAN (H-CRAN) architecture is composed of a small number of macro BSs and the large number of small BSs (micro, pico, femto) in combination with algorithms for the effective allocation of radio resources in centralized and integrated BBU pools is considered as a promising approach for minimizing network energy consumption. However, to support the expected increase in the number of UDs, wireless services and applications in the future, the number of network elements (the RRUs on the front-end side and the BBUs on the back-end side) will also increase, and the need for improving the RAN’s EE will remain.

One additional issue with C-RAN architecture is that all of its BBUs are always active, even when user traffic is low. This leads to the high energy consumption of the BBU pool. To address this issue, the authors in ref. [76] proposed a method for estimating BBUs’ resource-utilization rate. The method takes into account the data rate requirement, the number of mobile UDs, the RRU bandwidth and the transmission power between the RRU and mobile UDs (Table 3). They also presented the combine and remove (CnR) algorithm for deciding when to switch BBUs off and on. The proposed algorithms were developed with the aim of maximizing the number of sleeping BBUs while maintaining the QoS. The simulation results showed that when compared with traditional RANs, the proposed scheme can save the energy consumption of the BBU pool and the overall RAN system.

**Table 4:** Summary of the related works on PS methods in networks based on the MEC concept.

References	PS Method	Summary of Contributions Related to the Improvement of FiWi Network Energy Efficiency
[9]	Unified resource management scheme	The realization of the FiWi network with MEC led to a significant reduction in power consumption and an increase in the battery life of edge devices.
[91]	Unified resource management scheme and cloudlet-aware DBA algorithms	A resource management scheme that takes into account the use of cloudlets and incorporates offloading tasks into the FiWi DBA process. The proposed management scheme could significantly reduce the amount of energy used by edge devices and extend their battery life significantly.
[92]	Priority-based task offloading and caching (PrO) method	The proposed PrO scheme efficiently manages tasks by caching, offloading and performing local computing while preserving the priority order, which resulted in reduced delay and energy consumption.
[93]	ACCO and GT-CCO PS methods	Proposed a two cloud–MEC collaborative computation offloading mechanisms. Using a combination of MEC and centralized remote cloud services resulted in significantly lower energy consumption compared with solutions without a centralized cloud.
[94]	GT-CCO PS method	Proposed a FiWi network architecture that enables the coexistence of centralized cloud and MEC in the IoT applications. A game-theoretic collaborative computation offloading scheme was proposed as a solution for improving energy efficiency and handling a large number of mobile devices effectively.
[7]	ISA-CCO PS method	Confirmed that the proposed ISA-CCO solution is more effective than previously proposed ACCO and GT-CCO in terms of reducing energy consumption and improving processing response time on mobile devices.
[95]	TSGO PS method	Energy-efficient offloading strategy for MEC-enhanced FiWi. The three EE benchmarks to evaluate EE mechanisms for MEC FiWi were proposed and it was confirmed that the proposed strategy could significantly decrease energy consumption in MEC-enhanced FiWi networks.

Two methods for dynamic energy conservation in H-CRANs based on switching BBUs between the sleep mode and operational mode according to the changes in data traffic variations were presented in ref. [77] (Table 3). The first approach uses wake-on-local access network packets sent by the RRU to wake up BBUs. The second approach uses WoL packets to wake up BBUs sent to BBUs by the controller in the BBU pool. Both approaches were implemented and compared with respect to wake-up latency using software-defined (SD) radio prototypes, which were designed to be compliant with next-generation mobile networks (including 5G networks). To decrease energy consumption in C-RANs, the authors in ref. [78] proposed a joint resource-provisioning approach that leverages radio access unit (RRU) sleep scheduling and consolidation strategies of virtual machines hosting BBUs (Table 3). An efficient, low-complexity algorithm and a context-aware strategy were proposed to dynamically select active RRUs and virtual machines. The authors in ref. [78] proved that the proposed algorithm reduces the energy consumption of C-RANs.

In the C-RAN, the BBU pool serves a large number of RRU units (Figure 9) and by effectively coordinating the BBUs, network performance in terms of power consumption can be optimized. In order to minimize power consumption caused by communication overhead between the BBUs and RRUs, the authors in ref. [79] presented a scheme for the association of the BBU and the RRU based on graph partitioning and rejoining (Table 3). By using a partition and rejoin scheme, the authors assigned the BBUs to RRUs based on both the individual resource requirements of the RRUs and their communication with each other. The simulation results showed that the algorithm proposed in ref. [79] could reduce power consumption in the BBU pool with linear computational complexity.

In ref. [80], the authors presented a power-control algorithm that utilizes mobility prediction to improve EE in the 5G H-CRAN (Table 3). The proposed algorithm predicts the movement of user equipment in vehicular mobility situations and performs RRU switching based on the prediction results. The authors proposed an RRU-switching approach using Markov mobility prediction and a gradient-optimized transmission power method. The simulation results indicated that the proposed algorithm performed better regarding EE compared with existing RRU-switching algorithms.

Furthermore, the authors in ref. [81] developed a radio resource management method for energy-efficient H-CRAN (Table 3). The EE of H-CRAN was studied using an energy consumption model that includes all networking devices. Using this model, the authors developed an energy-efficient radio resource management algorithm to improve the network EE of the H-CRAN.

The authors in ref. [82] proposed an adaptive RoF system for reducing energy consumption while maintaining the required transmission rate the next-generation C-RAN systems (Table 3). The proposed system is based on the 2x2 MIMO–RoF model that employs coherent optical orthogonal frequency division multiplexing (CO-OFDM) technology. Through extensive numerical analyses, the authors verified that the proposed system could achieve significantly lower energy consumption with a high spectrum efficiency.

Additionally, the proliferation of the NFV technique has enabled the adoption of virtual machines which execute BBU functions in a way such that multiple virtual machines can run on a single, generic server at the BBU pool (Figure 9). By increasing the number of deployed RRUs and active virtual machines within the server, the authors in ref. [83] emphasized the need for solving the problem of increased power consumption. Therefore, the authors in ref. [83] proposed a power model that maximizes the network energy efficiency through the estimation of the optimal number of active virtual machines within the BBU server (Table 3).

To address the unsustainable growth of energy consumption caused by the increased mobile traffic, the C-RAN architecture separates the BBU from the RRH in the BSs and consolidates the BBUs into a BBU pool to allow low-utilized BBUs to enter sleep mode during decreased network activity, thereby reducing energy consumption. However, when a BBU is in sleep mode, the RRHs connected to it must be switched to another BBU, which can impact the QoS for those RRHs. To address this issue, the authors in ref. [84] have proposed a deep reinforcement learning (DRL) based BBU aggregation scheme that ensures both minimal energy usage of BBU and minimal migration of RRH traffic at the same time. Furthermore, several heuristic algorithms are introduced to assist with the DRL training. The proposed heuristic-assisted DRL (HA-DRL) approach is evaluated numerically and is found that the proposed approach outperforms the benchmarks by achieving the lowest cost for all scenarios. Additionally, the authors have found that the DRL agent is able to achieve better results by trading BBU power consumption for RRH traffic migration.

The utilization of C-RAN has proven to be effective in improving network performance. This gain is due to the smart management of RRHs with regard to power consumption and on/off operation modes. However, conventional resource allocation techniques maximize network efficiency without taking into account the overhead of RRH switching in adjacent time intervals. Therefore, in ref. [85], the authors aim to optimize EE while adhering to per-RRH transmission power and user data rate constraints. To achieve this, authors have formulated the EE problem as a Markov decision process (MDP) and have implemented DRL techniques to gain cumulative EE rewards. Simulation results showed that a proposed double deep Q network (DDQN)-based framework outperforms traditional approaches due to its ability to consider future effects of actions, and the ability to overcome the issue of action overestimation. This leads to a significant improvement in EE compared to benchmarks.

The presented analyses of related works indicate that many challenges related to improving EE in FiWI networks based on the C-RAN concept have not been addressed. It can be observed that further research activities related to the development of advanced architectures, signalizations, protocols and scheduling algorithms must be performed in order to make such FiWi networks more energy efficient.

## 5. Energy Efficiency Analyses of FiWi Networks Based on Multi-Access Edge Computing

**Figure 10:**
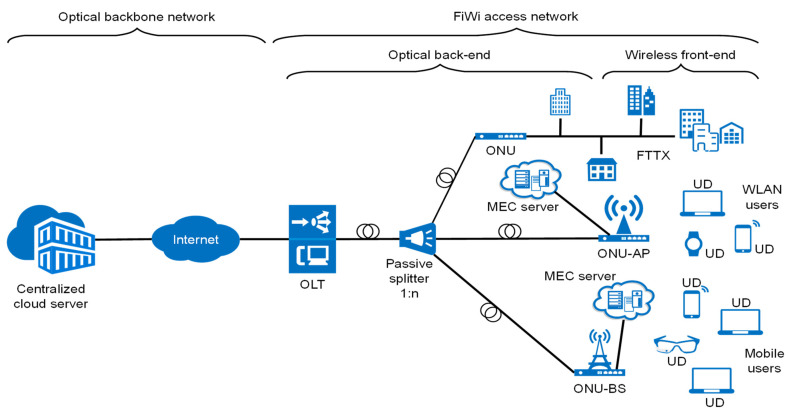
The generic architecture of a CM-FiWi access network.

The MEC concept introduces the cloud computing processes into FiWi networks, which are closer to mobile end UDs, more specifically at the mobile network edge. By exploiting an approach based on processing part of the user traffic in the cloud servers located at the edge of the mobile network, the transmission delay in the network is reduced and central servers at the cloud are less loaded. Figure 10 shows the generic network architecture that encompasses MEC and a centralized cloud paradigm over the FiWi access network. Such a network is referred to as a cloud–MEC FiWi broadband access network (CM-FiWi).

The architecture presented in Figure 10 consists of a long-reach optical backbone network connected to a centralized cloud and a standard FiWi access network supporting different wireless access technologies such as LTE, 5G or WLAN. One or more MEC servers are connected near the UDs of the fixed-access network or integrated with the ONU with the AP (ONU-AP) node or the ONU with base station (ONU-BS) node via a direct optical connection (Figure 10). This connection enables cloud services at the edge of the network, which can comprise the access of some wireless local/personal networks or cellular mobile networks (Figure 10). In such a FiWi network based on the MEC concept, ONU devices can also retain their traditional role, i.e., provide fixed services to users via the fiber-to-the-x (FTTx) concept (Figure 10).

This technology and architecture are key to the evolution towards full installation of the 5G networks and especially 6G networks, since it enables the transformation of mobile networks towards a programmable platform that meets the mobile cellular network requirements for increased bandwidth, lower delay and better scalability and configurability [96]. As one of the key technologies for enabling the full potential of 5G networks [97], MEC creates a pathway for the practical implementation of applications that require extremely low latencies with high reliability. Some examples of such applications include tactile internet, augmented reality/virtual reality, connected cars and mission-critical IoT systems. Moreover, the FiWi networks based on the MEC concept can reduce the overall operator CAPEX and OPEX through the exploitation of the existing infrastructure and through the implementation of integrated resource-management mechanisms.

The authors in ref. [9] reviewed the challenges and possible design scenarios for implementation of MEC-enabled hybrid FiWi networks with various RAN technologies (WLAN, 4G, LTE-A and HetNets) (Table 4). Additionally, using the TDMA scheduling resource-management scheme, the Ethernet-based FiWi network was further inspected regarding delay performance, battery life of edge UDs and response-time efficiency. The results showed that the MEC over FiWi can significantly reduce power consumption and extend the battery life of edge UDs (Figure 10). Furthermore, the same group of authors proposed, in ref. [91], a cloudlet-aware resource-management scheme that decreases the delay of offloading tasks and extends the battery life of edge UDs (Table 4). This scheme incorporates offloading the FiWi dynamic bandwidth allocation process and was designed using two TDMA layers to improve network performance. Analysis showed that the proposed solution, which incorporates cloudlets into the MEC FiWi networks, could lead to a significant reduction in energy usage for edge UDs and extend their battery life by several hours. The proposed architecture and resource-management strategy could be a useful solution for the implementation of MEC in future technologies such as the 5G tactile internet.

For the computational offloading scheme that involves moving intensive computing tasks to a cloud located at the edge of the mobile network is shown to be beneficial in improving EE and reducing latency for UDs in the mobile network [92]. Hence, the authors in ref. [92] proposed a priority-based offloading model that takes into account offloading and caching optimization that is combined with a local computation policy. The study shows that the proposed model has a substantial impact on decreasing both, the delay and energy usage in a cellular network.

Two collaborative computation offloading schemes using the CM-FiWi architecture, namely, an approximation collaborative computation offloading scheme (ACCO) and a game-theoretic collaborative computation offloading scheme (GT-CCO), were studied in ref. [93] (Table 4). The simulation results showed that using both MEC and centralized cloud services resulted in notably better energy efficiency of the network compared with the MEC schemes without centralized cloud offloading. Furthermore, in ref. [94], a generic FiWi architecture with a combination of centralized cloud and distributed MEC for IoT connectivity was presented (Table 4). The problem of collaborative computation offloading for the IoT over FiWi was addressed through the GT-CCO scheme. The numerical results showed that the proposed scheme was energy efficient and able to effectively handle a large number of mobile devices.

Due to limitations of mobile UDs, e.g., reduced computing resources, memory capabilities and limited battery capacity, offloading compute-intensive tasks to the MEC server or a remote cloud server emerged as a viable and promising solution for today’s computation and delay-sensitive applications [7]. This offloading scheme, called cloud–MEC collaborative computation offloading (CMCCO), takes advantage of both types of cloud services, i.e., centralized remote cloud service and a decentralized MEC service as two complementary technologies. The authors in ref. [7] proposed an energy-aware collaborative computation offloading (EA-CCO) system which can perform various computing tasks in the CM-FiWi network (Table 4). An iterative searching algorithm for collaborative computation offloading scheme (ISA-CCO) was developed in order to decrease task offloading overhead by taking into account residual battery rate, transmit power allocation and the scaling of computing resources. The simulation results showed that the proposed ISA-CCO solution achieved superior results compared with the aforementioned ACCO and GT-CCO paradigms in terms of energy consumption and processing response time of the mobile UDs.

Due to the removal of unnecessary data traffic from optical backbone networks, the MEC-enhanced FiWi network presents a viable choice for practical implementation in cases where resource-intensive and delay-sensitive mobile applications will be used.

However, the assumption that the FiWi infrastructure offers unlimited and free resources is not realistic and, hence, the energy-efficient offloading techniques must be considered in the realization of MEC-enhanced FiWi networks. Motivated by the need to develop energy-efficient offloading mechanisms for the MEC-enhanced FiWi networks, the authors in ref. [95] presented the two-layer Stackelberg game offloading (TSGO) strategy (Table 4). The FiWi layer and mobile edge computation offloading layer of this strategy are responsible for performing bandwidth allocation and offloading decisions, respectively. In addition, three EE benchmarks were proposed to evaluate the performance of the proposed strategy. The simulation results showed that the TSGO strategy supports the concept of green communications and could effectively decrease the energy usage of the MEC-enhanced FiWi networks.

## 6. Implementation of SDN-Based Energy Conservation Concepts in the FiWi Networks

In recent years, traditional access networks have become difficult to manage due to the constant increase in the number of users and network devices, which is followed by the increased complexity of the network structure. Compared with traditional communication networks, networks exploiting the SDN concept introduce flexible, dynamic and programmable networking concepts. The implementation of SDN results in easier network management and improved overall network performance [10]. The SDN allows for the virtualization of network functions (NFV) using applications that run on top of the SDN controller. These applications use a programming language that simplifies the rapid deployment of new services and capabilities. The SDN and its NFV applications can be used to manage the PON devices of different standards in a coordinated and dynamic manner [98].

**Table 5:** Summary of the related works on PS methods in SDN-based networks.

References	PS Method	Summary of Contributions Related to the Improvement of FiWi Network Energy Efficiency
[99]	SDN control mechanism through OpenFlow protocol	Confirmed that SDN-based control architecture has the potential to reduce energy consumption in the FiWi access network.
[100]	Enhanced standard PON devices with OpenFlow SDN technology and SD controller	An adaptive SD ONU PS mechanism that uses enhanced standard PON devices with advanced SDN capabilities. The simulation results showed that the proposed scheme could increase the EE while still guaranteeing the QoS requirements in a TDMA–PON system.
[98]	SD TWDM–PON architecture with OpenFlow technology	Development of the architecture that uses SD orchestration to coordinate wavelength/link speed deployment and to improve EE by adapting the link rate or activity state of the OLT/ONU transceivers during periods of low traffic while still maintaining the required QoS.
[101]	SDN-based 5G EPON architecture	Proposed an open control layer SDN-based framework that aims to minimize energy consumption in EPON while avoiding adding additional packet delay.
[102]	Controllers in OpenFlow technology	Energy-saving scheme for a FiWi access network that combines the OpenFlow technology in the SDN.
[103]	EEWA scheme	Proposed the EE scheme that significantly optimizes energy usage and workload allocation in a network combining the neighbor edge servers, local edge servers and the remote cloud. Proposed a path priority selection method to decrease the probability of network blocking and to improve the use of available spectrum.

**Figure 11:**
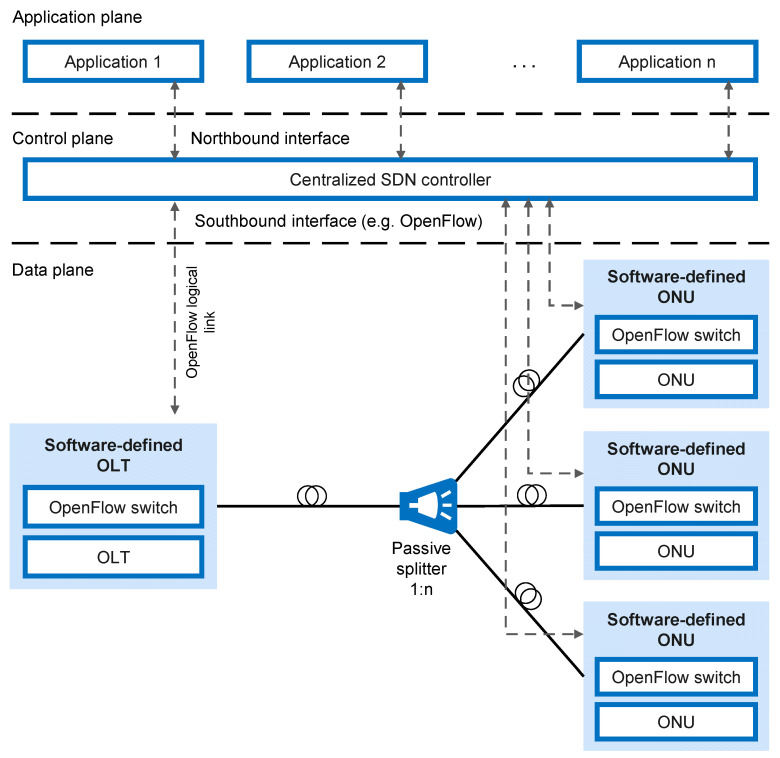
Software-defined PON architecture.

According to Figure 11, which presents a visualization of the SDN PON architecture, the separation of the data and control planes are the main features of SDN. Such separation enables centralized network management which is executed in the centralized controller. Two controller interfaces, namely, a northbound and southbound interface, are defined within the SDN architecture. They enable the centralized controller to communicate with applications and network equipment (Figure 11). The northbound interface is used to communicate with the application layer, which enables the application of SDN control management functions. On the other hand, the southbound interface is used for communication between a centralized controller located within the control plane and the data plane (Figure 11). The data plane consists of network equipment and it is responsible for packet-forwarding decisions. OpenFlow is a commonly used protocol at the southbound interface (Figure 11). It provides access to the data plane and is considered the enabler of the SDN concept [104].

Due to the constant network infrastructure growth, the power consumption of access networks has become one of the main concerns for companies that own larger networks and telecom operators. The SDN-based approach has emerged as a viable and effective solution for reducing power consumption of access networks [10]. Table 1 summarizes the related works on PS methods in SDN-based networks. The authors in ref. [99] showed through simulation that the proposed SDN-based energy management scheme in the EPON system can reduce the overall energy consumption of optical access networks (Table 5).

Although PON is considered an energy-efficient fixed network access technology, due to its mass deployment worldwide, PON systems need to be even more energy efficient to meet today’s green policy requirements. Due to modern consensual requirements related to ensuring more sustainable and energy-efficient operation of access networks, PON devices need to be enhanced in order to more easily adapt to such an environment. Having this as a goal, the authors in ref. [100] proposed an energy-conservation scheme through adaptive SDN-based TDMA–PON system architecture (Table 5). They introduced software-defined (SD)–, OLT– and SD–ONU architecture and employed an energy-saving scheme using an OpenFlow-based SD controller (Figure 11). To enable PS operation, the SD–OLT and SD–ONU devices were enhanced with SD agents connected to the SD controller via the OpenFlow protocol. The approach uses flow tables for packet classification and forwarding and an energy-saving table to store SD–ONU receiver sleeping periods. The transceiver of the SD–ONU was decoupled and thus the independent working state of the transmitter and receiver was enabled in such an architecture. Hence, depending on the traffic characteristics, the energy conservation of the SD–ONU was orchestrated via the SD controller through the assignment of the SD–ONU receiver’s sleep period and the SD–ONU transmitter’s wake-up threshold. The simulation results performed in low-traffic conditions showed a significant reduction in energy consumption, while maintaining QoS requirements.

In addition, an energy-efficient SD optical access architecture based on TWDM–PON was proposed in ref. [98] (Table 5). In this architecture, the authors enhanced existing fiber–optic network equipment with SD capabilities. SD orchestration was proposed to manage the use of different wavelengths and link speeds in the network. Through this management, saving energy could be achieved by turning off or decreasing the data rate of the OLT/ONU transceivers when traffic was low, while still maintaining the required quality of service. The authors in ref. [98] also evaluated the performance and EE of the proposed system in various scenarios which consider factors such as packet delay, link capacity and data loss in the network. The simulation results showed that various trade-offs should be considered when evaluating the EE of the system.

In order to minimize power consumption without adding additional delay in the 5G EPONs, the authors in ref. [101] proposed a framework based on the SDN and NFV paradigms (Table 5). The authors proposed an SDN 5G EPON network design that reduces the complexity of managing and operating the network, while improving the usage of network resources. The suggested solution extends the multipoint MAC control sub-layer in the OLT and divides its responsibilities between the OLT and the SDN controller. The management and control functions of the OLT and RAN were moved to the SDN controller, while the other functional components remained in the OLT and were integrated with the OpenFlow switch. Therefore, the functions that operate on longer time scales were transferred to the SDN controller, while those related to shorter time scales were kept in the OLT and RAN. This design allowed for the integration of EPON and RAN energy conservation techniques and additionally enabled the minimization of CAPEX costs. Nevertheless, the authors in ref. [101] emphasized the need for further evaluation of the proposed solution in order to clarify levels of energy savings.

The authors in ref. [102] proposed the PS scheme for FiWi networks based on an SDN approach (Table 5). The scheme is based on the OpenFlow protocol and a newly introduced controller that manages ONU’s working and sleeping states based on the traffic flow threshold. According to the simulation results, such a proposed centralized energy-saving approach based on SDN has a significant advantage. These advantages are mainly due to the central management provided by the introduced controllers. The controllers enable fair distribution of available resources, which results in greater energy savings. The authors demonstrated that the proposed SDN-based energy-saving scheme in the FiWi networks is effective and has practical value.

To achieve energy-efficient computing in a specific environment such as cloud-edge FiWi networks, it is crucial to coordinate the actions of edge servers and cloud servers in order to reduce energy consumption. Therefore, the authors in ref. [103] presented a solution for reducing energy consumption in such an environment by improving the cooperation between edge servers and cloud servers (Table 5). The proposed solution, called energy-efficient workload allocation (EEWA), was tested on an SDN testbed to demonstrate its feasibility. In addition, simulations were conducted to find the best possible outcomes for a set of task requests. The simulation results indicated that the EEWA scheme significantly decreased the network blocking probability and the average energy consumption in edge-cloud FiWi networks.

While implementation of SDN in RAN shows potential for improving EE of the FiWi networks, much work remains to be done within the areas of algorithms, standardization, architectures and interfaces.

## 7. Discussion

The development of new architectures, protocols and algorithms for FiWi networks has been a hot topic in the telecommunications industry in recent years. These networks combine the high speed and reliability of fiber optic cables with the convenience and accessibility of wireless technology, providing a cost-effective solution for delivering broadband services to a wide range of customers. Research on FiWi networking involves the integration of optical fiber and wireless broadband access technologies. FiWi broadband access networks resulting from the integration of optical fiber and wireless technologies can utilize both RoF and R&F hybrid networking technologies.

The carbon footprint of ICT infrastructure is gaining increasing public attention and raises concerns related to global warming. This has led to increased research into developing energy-efficient solutions and research dedicated to improving the EE of the FiWi networks is not an exception. The use of fiber optic cables, which transmit data using light signals, allows FWI networks to operate with lower power requirements than traditional copper wire or coaxial cable networks. This not only helps to reduce the energy costs of operating FWI networks, but also has environmental benefits, as it reduces the carbon footprint of telecommunications infrastructure.

In addition to the energy-efficient nature of fiber optic cables, FiWi networks can also incorporate other energy-saving technologies. In previous analyses presented for the RoF, R&F, MEC and SDN-based FiWi networks, it has been shown that advanced power-management schemes can be used to dynamically completely or partially turn off components in the FiWi network to reduce the power consumption of certain components when they are not in use.

While FiWi networks offer significant energy-saving potential, it is important for telecommunications companies to carefully consider the energy consumption of all aspects of FiWi networks, which includes the wireless components and the fixed network infrastructure required to support them. By adopting energy-efficient technologies and practices, service providers and telecom operators can significantly help to reduce the overall energy consumption of their FiWi networks and contribute to a more sustainable future.

Energy efficiency in C-RAN has become an increasingly important topic in recent years as the demand for mobile data services has continually grown. The C-RAN architecture, in which the baseband processing is centralized and the RF processing is distributed, has been proposed as a solution for improving energy efficiency in cellular networks. One of the main benefits of C-RAN is the ability to centralize baseband processing. By centralizing the baseband processing, the power consumption of C-RAN is reduced, since the centralized baseband unit (BBU) can serve more RRUs and thus can be designed to be more energy efficient in comparison with BBUs serving individual RRUs. Centralization also allows for the sharing of resources between BSs, which further contributes to reducing RAN power consumption. Furthermore, the C-RAN enables the dynamic power management of RRUs and BBUs by allowing them to sleep when the traffic is low, and wake up when the traffic demand increases. These BBU and RRU sleeping cycles contribute to the reduction of energy consumption.

Additionally, recent research studies proposed a wide range of methods for reducing energy consumption in the C-RAN. These methods include techniques for optimizing the use of resources in the BBUs, strategies for managing the sleep state of the BBUs, resource utilization algorithms for increased energy savings, approaches for associating BBUs and RRUs, power management algorithms of the RRUs and the use of NFV to execute the functions for improving the EE of BBUs using virtual machines. Therefore, C-RAN architecture has the potential to significantly improve the energy efficiency of cellular networks through centralization, fronthaul compression, dynamic power management of radio resources and the use of the SDN and NFV. However, careful planning is required to ensure that the high bandwidth requirements of the fronthaul links can be met and to avoid adding complexity to the network.

In the presented analyses, it is shown that EE is an important concern in the design and operation of the FiWi networks due to the fact that the energy consumption of these networks can be a significant portion of the total OPEX of network operators. One way to improve the EE of FiWi networks is to use MEC technology. The MEC allows the deployment of computing and storage resources closer to the edge of the network and thus closer to the users. This can reduce the energy consumption of the network by reducing the amount of data that needs to be transmitted over long-distance fiber–optic links between remote cloud servers and users located at the edge of the wireless network, since more data processing can be carried out locally at the edge servers. Optical fibers are already recognized for their EE and, implemented with MEC technology, can additionally diminish unneeded data transfer in optical networks. However, energy efficiency in the MEC-based FiWi networks is still largely unexplored and it should be a highly prioritized subject of future research.

In addition, energy-efficient techniques for MEC-enabled FiWi networks include power-aware resource management schemes. These schemes optimize the allocation of resources to the fiber–optic and wireless links based on the traffic demand and the available energy. This optimization can be performed in combination with collaborative computation offloading schemes, which computationally redirect demanding tasks from the central remote server in the cloud to a server located closer to the users at the edge of the network. These concepts have the potential to contribute to the full proliferation of the green communications paradigm and to significantly decrease energy consumption in the FiWi networks.

One advantage of using the SDN in FiWi networks is the ability to dynamically control and optimize network resources. With the SDN concept, network administrators can use a centralized controller to manage and configure network devices, which allows the simple allocation of resources and the adjustment of network configurations as needed. Such an SDN concept can also help to improve the efficiency and performance of FiWi networks, particularly in dynamic or high-traffic-demand environments. Because network configurations can be easily adjusted using the centralized controller, it is easier to add or remove devices and services as needed. This will enable telecommunication providers to satisfy the changing needs of their customers and adaptation to new technologies and trends. Overall, the use of SDN in the FiWi networks can help to improve the efficiency, performance and flexibility of these networks, making them a more attractive option for telecommunications providers and their customers.

In conclusion, FiWi networks offer a cost-effective and energy-efficient solution for delivering broadband services to a wide range of customers. By leveraging the energy-saving potential of fiber optic cables and incorporating other energy-efficient techniques and strategies, telecommunications providers can help to reduce the environmental impact of their networks and contribute to a more sustainable future. The use of energy-efficient techniques presented in this survey can significantly improve the EE of FiWi networks, thus reducing their operating costs and carbon footprints.

## 8. Conclusions

This paper presented a comprehensive survey of recent research on approaches that contribute to energy efficiency improvements of FiWi access networks. Emphasis was given to the extensive literature review of various power-saving techniques and energy-efficient models that are dedicated to the improvement of FiWi network energy efficiency. The presented review covered energy efficiency analyses of different types of FiWi networks which include the R&F FiWi networks, the RoF FiWi networks, the MEC-based FiWi networks and the FiWi networks based on the SDN concept. For the R&F networks, energy conservation techniques and research studies related to the optical and wireless domains were presented, as well as related works that deal with the improvement of FiWi networks’ energy efficiency through the cooperation of techniques in wireless and optical domains. Furthermore, two basic RoF techniques, the D–RoF and A–RoF, were elaborated in the context of energy efficiency, and an overview of research studies in the field of improving the energy efficiency of D–RoF and A–RoF systems was given. Additionally, the C-RAN architecture was reviewed through the prism of energy consumption with the presentation of current research efforts related to the improvement of the C-RAN energy efficiency. The MEC-based FiWi networks, which introduce cloud computing at the edge of the mobile network, were further presented, and articles dedicated to the mechanisms and concepts for the optimization of the MEC FIWi network’s energy consumption were highlighted. Finally, flexible SDN FiWi networks that offer high scalability and ease of management of the FiWI networks were presented, with an emphasis on research related to energy conservation techniques implemented in such networks. The literature suggests that there are many areas in which the energy efficiency of the FiWi networks can be enhanced. Overall, the overview presented in this work showed that EE is one of the major concerns in the FiWi networks and further intensive research attempts should be carried out in the endeavor of improving the energy efficiency of the FiWi networks.

## Abbreviations

100G-EPON	100 Gbit/s Ethernet Passive Optical Network
10G-EPON	10 Gbit/s Ethernet Passive Optical Network
3G	3rd Generation Mobile Network
4G	4th Generation Mobile Network
5G	5th Generation Mobile Network
6G	6th Generation Mobile Network
ACCO	Approximation Collaborative Computation Offloading
ALR	Adaptive Link Rate
ANN	Artificial Neural Network
AP	Access Point
A–RoF	Analog Radio-over-Fiber
AWSM	Adaptive Watchful Sleep Mode
BBU	Baseband Unit
BS	Base Station
CA	Carrier Aggregation
CAPEX	Capital Expenditure
CMCCO	Cloud–MEC Collaborative Computation Offloading
CM-FiWi	Cloud–MEC FiWi
CnR	Combine and Remove
CO	Central Office
CoMP	Coordinated Multi-Point
CO-OFDM	Coherent Optical Orthogonal Frequency Division Multiplexing
CPRI	Common Public Radio Interface
C-PSM	Centralized Power-Saving Mode
C-RAN	Cloud Radio Access Network
DAS	Distributed Antenna System
DBA	Dynamic Bandwidth Allocation
DCF	Distributed Coordination Function
DDQN	Double Deep Q Network
DEC	Delay-Controlled and Energy-Efficient Clustered
D-RAN	Distributed Radio Access Network
DRL	Deep Reinforcement Learning
D–RoF	Digitized Radio-over-Fiber
DRP	Dynamic Resource Provisioning
DWBA	Dynamic Wavelength and Bandwidth Allocation
E/O	Electrical-to-Optical
E/O/E	Electrical-Optical-Electrical
EA-CCO	Energy-Aware Collaborative Computation Offloading
ECO-FiWi	Energy Conservation Scheme for FiWi Networks
EE	Energy Efficiency
EEWA	Energy-Efficient Workload Allocation
EPON	Ethernet Passive Optical Network
FiWi	Fiber–Wireless
FTTA	Fiber to the Antenna
FTTx	Fiber to the x
GA	Genetic Algorithm
GDBA	Green Dynamic Bandwidth Allocation
G-EPON	Gigabit Ethernet Passive Optical Network
GHG	Greenhouse Gas
GPMM	Generic Power Management Model
GPON	Gigabit Passive Optical Networks
GT-CCO	Game-Theoretic Collaborative Computation Offloading Scheme
HA-DRL	Heuristic-Assisted Deep Reinforcement Learning
H-CRAN	Heterogeneous Cloud Radio Access Network
HERM	H-CRAN Energy-Efficient Radio Resource Management
HetNet	Heterogeneous Network
ICI	Inter-Cell Interference
ICT	Information And Communications Technology
IFoF	Intermediate Frequency-over-Fiber
ILP	Integer Linear Programming
IoT	Internet Of Things
ISA-CCO	Iterative Searching Algorithm for Collaborative Computation Offloading
IT	Information Technology
LoRa	Long Range
LPWAN	Low-Power Wireless Access Network
LTE-A	Long-Term Evolution Advanced
M2M	Machine-to-Machine
MAC	Media Access Control
MANET	Mobile Ad Hoc Network
MDP	Markov Decision Process
MEC	Multi-Access Edge Computing
MFH	Mobile Fronthaul
MFN	Mobile Fronthaul Network
ML	Machine Learning
mMIMO	Massive Multiple-Input Multiple-Output
mmWave	Millimeter Wave
MNO	Mobile Network Operator
MN	Mobile Network
MPP	Mesh Portal Point
NB-IoT	Narrowband Internet of Things
NFV	Network Function Virtualization
NG–PON2	Next-Generation Passive Optical Network 2
OBSAI	Open Base Station Architecture Initiative
OLT	Optical Line Terminal
ONU	Optical Network Unit
ONU-AP	Optical Network Unit with Access Point
ONU-BS	Optical Network Unit with Base Station
OPEX	Operating Expenditure
ORI	Open Radio Equipment Interface
OSC	ONU Sleep Controller
OSI	Open Systems Interconnection
PON	Passive Optical Network
PrO	Priority-based task offloading and caching
PS	Power-Saving
PSM	Power Saving Mechanism
PSO	Particle Swarm Optimization
QoS	Quality Of Service
QPSO	Quantum Particle Swarm Optimization
R&F	Radio-and-Fiber
RAN	Radio Access Network
RAU	Remote Access (Antenna) Units
RF	Radio Frequency
RFoF	Radio Frequency-over-Fiber
RI	Radio Interface
RoF	Radio-over-Fiber
RRU	Remote Radio Unit
SAPSM	Smart Adaptive Power Save Mode
SD	Software-Defined
SDN	Software-Defined Network
SEMC	Save Energy and Maximize Connectivity
SIEPON	Service Interoperability in the Ethernet Passive Optical Networks
TDM	Time-Division Multiplexing
TDMA	Time Division Multiple Access
TRx	Transceiver
TSC	Tx Sleep Controller
TSGO	Two-Layer Stackelberg Game Offloading
TWDM	Time And Wavelength Division Multiplexing
Tx	Transmitter
WDM	Wavelength-Division Multiplexing
WLAN	Wireless Local Area Network
WMN	Wireless Mesh Network
WOTR	Wireless–optical Topology Reconfiguration
WSM	Watchful Sleep Mode
